# Expanding options for HIV testing: A process evaluation of a community-led HIV self-testing intervention among men who have sex with men in Kenya

**DOI:** 10.12688/gatesopenres.14819.1

**Published:** 2023-10-20

**Authors:** Memory Melon, Bernadette Kombo, Mary Mugambi, Margaret Njiraini, Kennedy Olango, Manas Migot, Samuel Kuria, Martin Kyana, Peter Mwakazi, Japheth Kioko, Shem Kaosa, Maria Mensah, Matthew Thomann, Janet Musimbi, Helgar Musyoki, Parinita Bhattacharjee, Robert Lorway, Lisa Lazarus

**Affiliations:** 1 Partners for Health and Development in Africa, Nairobi, Kenya; 2 Institute for Global Public Health, University of Manitoba, Winnipeg, Manitoba, R3E 0T6, Canada; 3 National STI and AIDS Control Programme (NASCOP), Ministry of Health of Kenya, Nairobi, 00202, Kenya; 4 Men Against AIDS Youth Group (MAAYGO), Kisumu, Kenya; 5 Mambo Leo Peer Empowerment Group (MPEG), Kiambu, Kenya; 6 HIV & AIDS People's Alliance of Kenya (HAPA- K), Mombasa, Kenya; 7 Department of Anthropology, University of Maryland, College Park, Maryland, 20742, USA

**Keywords:** HIV, self-testing, MSM, community-based organizations, Kenya

## Abstract

**Background:**

Men who have sex with men (MSM) in Kenya continue to face barriers to HIV testing, which leads to delays in HIV prevention and care. An HIV self-testing (HIVST) intervention was implemented in three Kenyan counties to increase coverage and frequency of HIV testing among MSM communities with high HIV prevalence. The evaluation study examined how HIVST can increase testing among MSM who are unaware of their status by increasing coverage, frequency, and early uptake of testing and support linkages to prevention and treatment. We share results from the process evaluation of the intervention implemented in partnership with MSM-led organizations.

**Methods:**

For a 12-month period between August 2019 and July 2020, the project team conducted in-depth interviews with HIVST users, monthly meetings with programme implementation teams, and monthly monitoring data reviews. Polling booth surveys were also conducted with participants. The process evaluation explored the fidelity, feasibility, coverage, acceptability, quality, and effectiveness of the HIVST intervention.

**Results:**

An average of 793 MSM received 1,041 HIVST kits on a monthly basis through different distribution channels. Of those who received HIVST kits, 67% were distributed to infrequent testers and non-testers. Testing frequency among users increased to 82% for those who had a recent test during the previous three months, compared to 58% of HIVST non-users. There was a high linkage to care and treatment services (84%) among those who tested reactive for HIV at endline. MSM shared preferring HIVST kits because of its convenience and privacy. During the COVID-19 pandemic, adaptations to the intervention were made to support ongoing HIV testing and linkages to services.

**Conclusion:**

The introduction of HIVST in MSM-led HIV prevention programmes was feasible with high acceptability. The involvement of the MSM community in the design, implementation and evaluation of the intervention was a key factor to intervention success.

## Background

HIV testing is a necessary first step in the HIV care continuum ( Nnko *et al.*, 2019). Knowing one's HIV status provides a vital opportunity to engage in HIV care and treatment ( Nnko *et al.*, 2019; WHO, 2016). Awareness of one's HIV status also impacts HIV prevention behaviors, such as consistent condom use ( Venkatesh *et al.*, 2011). The World Health Organization recommends HIV testing and counseling as part of comprehensive HIV prevention and treatment services for key populations ( WHO, 2016), but access to HIV testing among these populations remains a challenge. In a study among transgender women and men who have sex with men (MSM) in sub-Saharan Africa, nearly all participants (92.2%) stated that they had previously taken an HIV test, but more than a third (42.1%) reported more than six months since their most recent test ( Sandfort *et al.*, 2019).

HIV testing is a key component of the key population programme in Kenya and the official guidelines recommend quarterly testing for MSM ( National AIDS and STI Control Programme, 2014a). However, the country’s existing routine monitoring data show significant gaps in service uptake throughout the treatment cascade ( DHIS2, 2021). The National AIDS and STI Control Programme (2014b) indicated that 18.2% of MSM were living with HIV, with service coverage at 65% ( National AIDS Control Council, 2018). Treatment programme outcomes showed that 52% of MSM living with HIV knew their HIV status, 80% of MSM living with HIV received antiretroviral therapy (ART), and 74% of those who received ART demonstrated viral suppression ( Musyoki *et al.*, 2021). According to Hlongwa *et al.* (2020), a high level of stigma and discrimination makes it difficult for MSM to access health care services, while Okall and colleagues (2014) point to challenges related to stigma and concerns about disclosing one’s sexual orientation and confidentiality as ongoing challenges for MSM in accessing services in Kenya. These access barriers highlight the need for more targeted and strategic testing services, accessibility to a variety of HIV testing choices, and stigma-free services in order to achieve UNAID’s goal to halt the AIDS epidemic by 2030 ( UNAIDS, 2015).

Studies have shown that HIV self-testing (HIVST) is a potential gateway to improving testing rates among MSM ( Zhang *et al.*, 2017). In studies conducted in Kenya, men were willing and ready to use HIVST ( Thirumurthy *et al.*, 2016). The National AIDS and STI Control Programme (NASCOP), in collaboration with three MSM-led community-based organizations (CBOs) in Kenya, namely the Mamboleo Peer Empowerment Group (MPEG) in Kiambu, Men Against AIDS Youth Group (MAAYGO) in Kisumu, and the HIV & AIDS People's Alliance of Kenya (HAPA Kenya) in Mombasa, the University of Manitoba, and Partners for Health and Development in Africa (PHDA), implemented an intervention with the main objectives of increasing testing coverage and frequency and fostering early linkage to prevention, care, and treatment through demand creation, service delivery, and follow-up. The intervention provided HIVST as an alternative HIV testing method during a 12-month period. The evaluation study examined how HIVST can increase HIV testing among MSM who are unaware of their HIV status by increasing coverage, frequency, and early uptake of HIV testing and support linkages to prevention and treatment among those who use HIVST. This article outlines the process of putting the interventions into practice in the three counties, with an emphasis on demand generation approaches, service delivery mechanisms, and service connections. Additionally, we share results from the process evaluation exploring the fidelity, feasibility, coverage, acceptability, quality, and effectiveness of the HIVST intervention.

## Methods

### Study setting

The study was conducted in Mombasa, Kisumu, and Kiambu counties, where the key population mapping and size estimation performed by NASCOP in 2018 indicated a total of 2,855, 2,492, and 1,664 MSM, respectively ( National AIDS and STI Control Programme, 2019). According to a virtual mapping survey conducted in the same counties, 25% of MSM operated virtually rather than visiting actual physical locations to find sexual partners ( Emmanuel *et al.*, 2020). As of July 2020, HAPA Kenya in Mombasa had registered 2,545 MSM, MAAYGO in Kisumu had registered 2,432 MSM, while MPEG in Kiambu registered 2,166 MSM. As per data from a polling booth survey conducted in 2017, the self-reported HIV prevalence among MSM was 19% in Mombasa, 13% in Kisumu, and 23% in Kiambu County ( National AIDS and STI Control Programme, 2018).

### HIVST intervention

The conception and execution of the HIVST programme was guided by the 2017 HIV Self-Testing Guidelines, the 2018 Young Key Population Guidelines, and the 2014 Key Population Guidelines from NASCOP, which members of our team were instrumental in developing with the Ministry of Health of Kenya. The intervention, which began concurrently in all three sites between August 2019 and the end of July 2020, was integrated into already-existing HIV and sexual health programmes carried out by the three partner CBOs in their respective counties with the goal of reaching all MSM over the age of 15 ( Bhattacharjee *et al.*, 2019).

To guide the design of the intervention, baseline quantitative and qualitative research was carried out. The HIVST implementation team, which included programme managers, programme officers, field officers, outreach workers and peer educators from the three CBOs were part of the intervention design with a focus on demand generation and mobilization, service provision, and connection to prevention and treatment-related services. Under the supervision of 19 outreach workers, a total of 161 peer educators were engaged in the intervention process as the main team. Each month, outreach workers, project staff, and regional field coordinators from PHDA met with the peer education teams in Mombasa, Kisumu, and Kiambu, numbering 63, 60, and 38 peer educators, respectively, to discuss the success of the interventions and to develop recommendations. The programme management team consisted of three study coordinators, five clinicians, four HIV testing services (HTS) counselors, and four monitoring officers.

MSM who test infrequently and non-testers were the main target groups for the distribution of HIVST kits. "Frequent testers" were defined as MSM who had an HIV test during the previous six months; "infrequent testers" were those who had an HIV test within the previous six to twelve months; and "non-testers" were MSM who had not had an HIV test within the previous twelve months.

### Demand creation and mobilization

**Figure 1.**
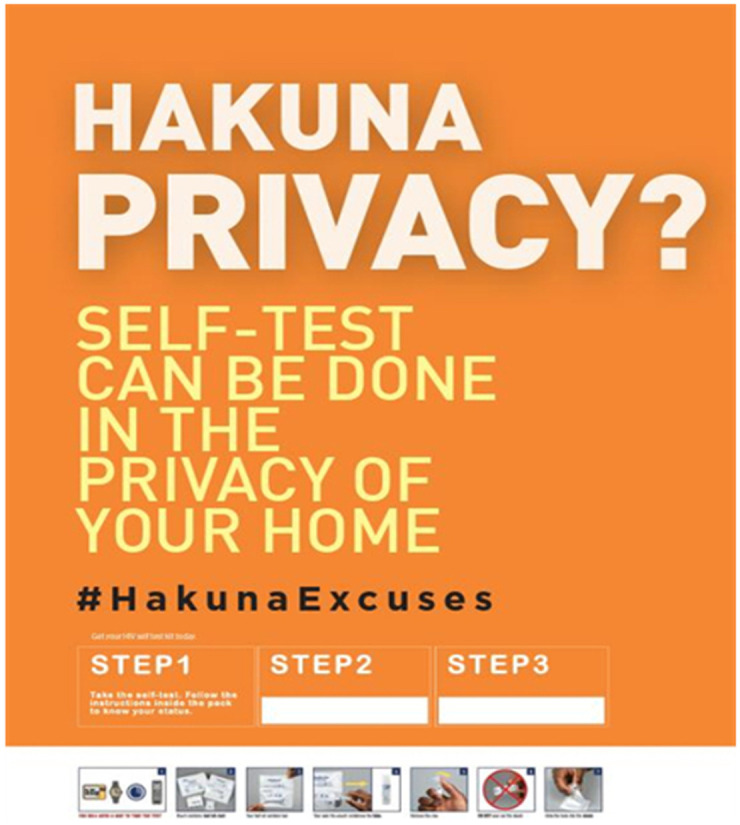
Poster to create awareness on HIVST.

Peer educators created demand for HIVST through hotspot-based peer connections, during key population drop-in centre (DIC) events at the organizations, and at promotional events for HIVST. Online demand creation was also utilized through channels such as Facebook, WhatsApp groups, and MSM-specific dating sites such as Grindr, Planet Romeo, and Hornet. Across the three sites, an average of 4,659 MSM were contacted each month from both physical and virtual venues. In order to create demand, health care providers in the three sites provided information about HIVST at the DICs and during clinical outreaches. The peer outreach workers also used physical and electronic posters with HIVST messages that highlighted the advantages of HIVST kits and provided access to more resources, as well as t-shirts, pamphlets, banners, aprons for bartenders, reflectors for riders' safety, and posters in the restrooms of club-based hotspots (see Figure 1– Figure 3).

**Figure 2.**
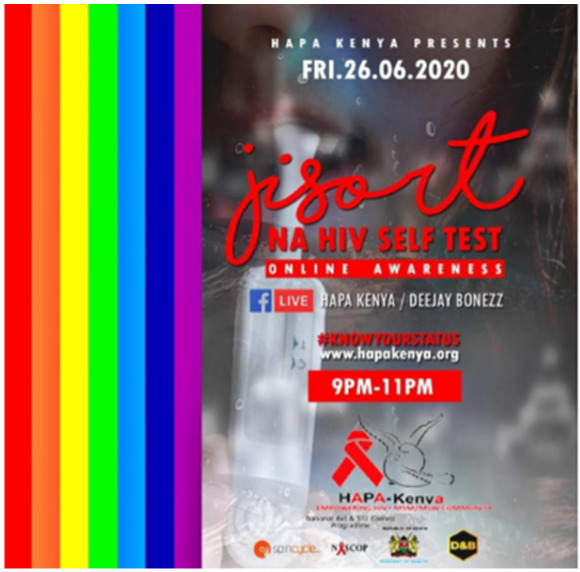
Poster for virtual promotion of HIVST.

**Figure 3.**
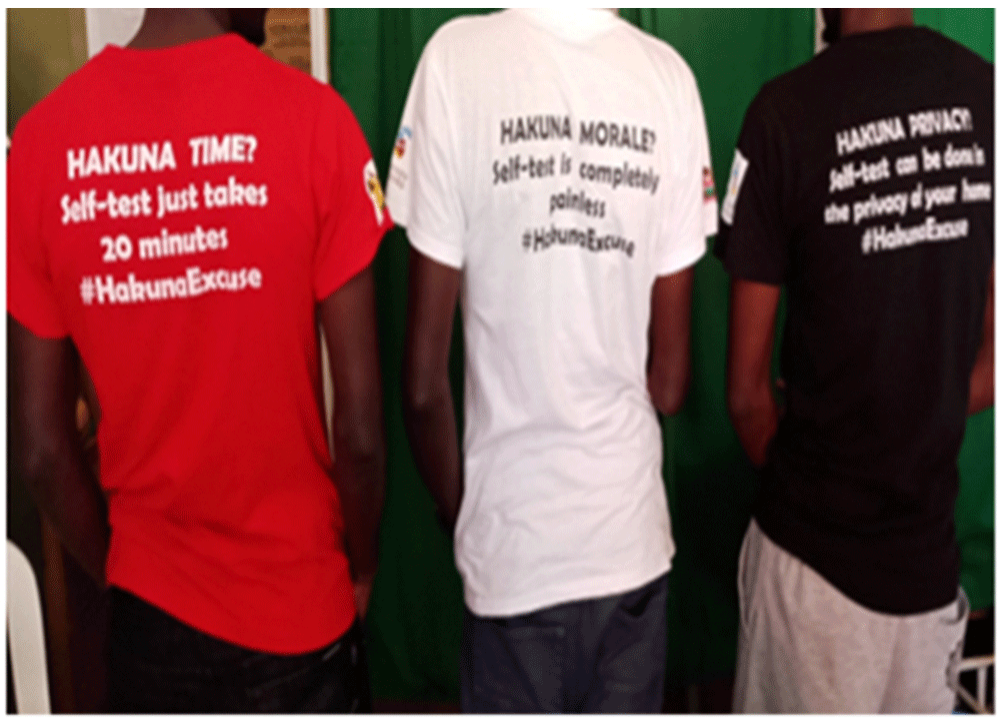
Printed t-shirts with messages to create demand and awareness for HIVST.

A total of 18 social events were planned by peers across the sites, including DJ nights, MSM-themed parties, fun days (which featured social events like coffee dates, fashion displays, and grooming), World AIDS Day celebrations, and "Let's Get Real" PrEP-related gatherings. These events reached 3,227 MSM on average (78% from physical places; 22% from virtual spaces) with HIVST messages each month.

### Service delivery

HIVST was made available to MSM in the three counties through two main delivery channels: (i) community-based delivery channels and (ii) facility-based delivery channels. A trained group of individuals, including clinicians, nurses, outreach workers, peer educators, and peers, delivered the kits. Along with the kits, the team provided information and education about sexuality, safer sex practices, HIV testing services, and prevention and treatment services to meet the needs of people who were not enrolled in programmes.

***Community-based delivery channels***. This approach saw HIVST kits being promoted and distributed by and within the MSM community. Within this channel of distribution, two methods were used to offer kits to the community *.* Direct methods entailed trained MSM peer outreach workers distributing HIVST kits directly to their peers during peer outreaches at hotspots, during events such as parties, clubs, and DIC gatherings. Indirect methods involved the distribution of kits through social and sexual networks, where peers who were offered HIVST kits could collect additional kits to offer to their networks. The goal of indirect distribution was to reach people who were not enrolled in or served by the programmes ( Bhattacharjee *et al.*, 2019). The Kisumu site relied on peers described as "super mobilisers" (peers who were known to have extensive social and sexual networks) for distribution. Indirect distribution channels for MSM, additionally included delivery to peers' homes and the use of courier services, which was commonly used among virtual peers.

***Facility-based delivery channels***. This distribution channel aimed at offering HIVST kits within health care settings. MSM were able to obtain HIVST kits directly from key population programme clinics and DICs, including those integrated within government facilities. In these settings, health care providers offered HIVST kits to MSM during their routine visits to the clinics. MSM were provided with a dedicated room or space to perform the test or would otherwise be assisted by a health care provider or carry the kit home for later testing. Additionally, MSM were provided with HIVST kits during regularly organized clinical outreaches, which occurred in convenient locations, such as hotspots. In all cases, MSM were offered the choice of assisted (supported by outreach or clinical professionals) or unassisted (on their own) self-testing based on their preferences.

### Linkage to HIV prevention and treatment services

A coordinated follow-up system was put in place a few months into the intervention to efficiently help connect the recipients of HIVST kits to prevention and treatment services. For MSM who had consented to be followed, the clinical team (HTS providers and clinicians) made phone calls and sent text messages, with the assistance of peer educators. Peer educators and peers supported follow-up for MSM not consenting to be followed by health care providers. Referral cards with clinical contacts were given out along with HIVST kits so that participants could call or text the clinical team for more information or for referrals. To facilitate linkages to services, accompanied referrals were also offered.

### COVID-19 adaptations

The COVID-19 pandemic occurred during the intervention, necessitating a re-strategizing of the project. The team scaled up virtual demand generation through the use of virtual mobilization and online HIVST communication materials, such as short messages, videos, YouTube sensitizations, and Facebook DJ nights. Additionally, HIVST kits and HIV prevention commodities were delivered using motorcycles, mobile vans, door-to-door delivery, and courier services, along with messages promoting HIVST and COVID-19 prevention. HIVST kit distribution was expanded to include frequent testers who could not get to DICs or other health institutions for routine testing because of lockdowns and curfews. Follow up of HIVST users was intensified and any HIVST user experiencing symptoms such as fever, cough or difficulty in breathing were advised to seek medical care.

### A process evaluation of the HIVST intervention

To evaluate the HIVST intervention, the research team employed a mixed-methods approach.

*Longitudinal qualitative cohort:* The longitudinal study assessed the effectiveness of different delivery mechanisms on improving the coverage and frequency of HIV testing. Community researchers developed a sampling plan to enroll 24 participants from each of the three sites. Participants were age 18 years and older, had sex with other men in the last 12 months, assigned male sex at birth, and willing to be followed over a period of one year for in-depth interviews at baseline, midline and at endline. Attention was paid to ensure representation of different ages, socio-economic status, and involvement in sex work, as well as experiences of MSM who were enrolled in CBOs compared to those who were not. Interviews were conducted by community researchers who underwent a four-day training workshop on research ethics ( Anderson, 2011) and qualitative research methods. Community researchers conducted the interviews in the language most comfortable to the participants in safe spaces run by the community-led organizations and in selected venues which were identified by the peer educators in consultation with the community and considered to be secure and MSM-friendly. Interviews lasted approximately an hour and were recorded, transcribed, and translated (where necessary). Seventy baseline, 36 midline, and 46 endline in-depth interviews were conducted with HIVST service users. Community researchers were then involved in participatory thematic analysis of the transcripts ( Kombo *et al.*, 2023).

*Meeting reports:* Monthly routine monitoring reports using standard reporting tools were produced by the outreach and clinical teams and were analyzed at the site-level on a monthly basis, and shared with the implementation team to discuss areas for improvement. A more detailed analysis was conducted by the implementation team quarterly to assess emerging themes.

*Polling booth surveys*: In order to understand progress towards achieving the intervention's goals and to inform mid-course adjustments in the design of the intervention, we conducted a population-based quantitative assessment using a polling booth survey (PBS) at baseline and endline of the project, as suggested by Behanzin *et al.* (2013), and included further propositions on the study design according to Bhattacharjee *et al.* (2019). PBS operates as an anonymous group interview to minimize social desirability bias and offers a low cost and rapid assessment process.

The initial round of PBS was executed in Kiambu, Kisumu, and Mombasa in September 2019, with the endline surveys taking place in August 2020. These surveys were carried out by programme staff, outreach workers, and peer educators from three community-led CBOs, receiving technical and supervisory assistance from the NASCOP Technical Support Unit. Participants for the PBS were selected randomly by peer educators from a list of sampled physical and virtual hotspots. Participants were subsequently invited for interviews at venues determined through consultation with the peer educators. A significant proportion of the surveys took place within the secure settings of the DIC's safe spaces and board rooms, while the remaining interviews were conducted in safe and secure locations within hotspots, particularly for individuals located at a distance from DICs. The survey took around 45 minutes and collected information on the coverage and frequency of HIV testing and HIVST, the use of different delivery mechanisms, and linkages to prevention and treatment after testing, exploring questions, such as “Did you take an HIV test during the past 3 months? If yes, did you use HIV self-testing? Are you living with HIV? If yes, are you registered in an HIV care and treatment programme?”. In total, 1,497 participants participated in the PBS surveys at baseline and endline.

Additionally, we adopted Busza and colleagues’ process evaluation framework, previously used to evaluate an intervention for young women who sell sex in Zimbabwe ( Busza *et al.*, 2016). Applying this framework to our data, we evaluated the fidelity (the degree to which the intervention was delivered as intended), feasibility (the difficulties encountered and how they were resolved), coverage (the degree to which the programme reached target participants), acceptability (participants' perceptions of the intervention), quality (how well the interventions were delivered), and effectiveness (did the intervention lead to change) of our HIVST programme.

### Ethics

Ethics approval was obtained from the institutional review boards of the Kenyatta National Hospital – University of Nairobi, Kenya (P557/08/2018) (December 6, 2018) and the University of Manitoba – Health Research Ethics Board, Canada (HS22205) (January 8, 2019).

### Consent

Written informed consent was obtained from all participants prior to their participation in the study. Participants aged 15+ years were considered mature minors, in alignment with the national HIV testing guidelines ( National AIDS and STI Control Programme (NASCOP) & Kenya Medical Research Institute (KEMRI), 2015), which stipulate the minimum age for HIV testing without parental/guardian consent as 15 years and approved by ethics boards in Kenya and Manitoba.

## Results

### Programme outcomes

In each site, an average of 793 MSM received 1,041 HIVST kits per month during the course of the 12-month intervention of which 86% and 14% were distributed through community and facility channels, respectively. Sixty-seven percent of the kits were given to infrequent testers and non-testers. In total, 3,989 MSM visited the clinic for prevention and treatment services for follow-up during the intervention period. In the section below, findings are examined using the process evaluation framework developed by Busza *et al.* (2016) in order to better understand the programme's results (summarized in Table 1).

**Table 1.** Process evaluation questions and outcomes *.

Measure	Key questions	Data Source	Findings
Fidelity	Were key activities delivered?	-Monthly meeting reports	-Demand creation strategies worked well to promote the use of HIVST kits. -MSM were reached in both physical and virtual spaces with HIVST.
Feasibility	What challenges were encountered?	-Monthly meeting reports -Midline and endline in-depth interviews	-Minimal challenges experienced. -Concerns related to follow-up of HIVST users for prevention and treatment services, especially peers who received kits through secondary distribution channels.
Coverage	How many target participants were reached?	-Monthly monitoring reports -Monthly meeting reports	-An average of 793 MSM received 1,041 HIVST kits on a monthly basis from the three sites. -67% of the kits were distributed to infrequent testers and non-testers.
Acceptability	What were the community perceptions and did they actively participate in the project?	-In-depth interviews -Monthly meeting reports	-MSM actively and meaningfully contributed in the design, implementation, and monitoring of the intervention. -The majority of MSM received and used the HIVST kits. They had a positive view of the intervention and preferred HIVST due to increased confidentiality, choice, and convenience.
Quality	Are the activities appropriate and evidence- informed?	-Monthly meeting reports	-The choice of activities was considered appropriate. -Virtual platforms created an opportunity to discretely reach MSM through virtual peer educators.
Effectiveness	Are the intended outcomes occurring?	-Polling Booth survey -In-depth interviews	-31% of MSM from physical and virtual sites used HIVST kits in the past three months at baseline, which increased to 47% at endline. -63% of MSM received services from the project intervention in the last three months at baseline, which increased to 73% at endline. - Increase in new diagnoses from 13% at baseline to 19% at endline.

* Adapted from Busza *et al.*, 2016

### Fidelity

The process evaluation showed fidelity to demand generation, service delivery, and linkage to prevention and treatment activities and timelines prior to the COVID-19 pandemic. HIVST was effectively communicated to MSM from both physical hotspots and virtual platforms thanks to the participation of both types of peer educators. The approaches used for creating demand were successful in encouraging the use of HIVST kits. While the promotional messages emphasizing the simplicity of HIVST were successful, participants shared that they found them to be a little too repetitive, especially for the virtual mobilization. Although infrequent and non-testers were the target MSM sub-populations for HIVST, COVID-19 shifted focus to also include frequent testers.

### Feasibility

Prior to the intervention, all teams received training to support the successful rollout of the intervention ( Kombo *et al.*, 2023). Peers raised concerns surrounding confidentiality, including how the project staff would be able to follow-up with HIVST users for prevention and treatment while maintaining confidentiality. As a result, each of the three participating sites built a central follow-up mechanism. The clinical team was entrusted with monitoring all willing users of the HIVST kit. The peer educators reported that, men who had not declared their MSM status were more hesitant to visit the clinic after testing because they feared being identified as MSM.

Based on the intervention's design and the national HIV testing protocol, confirmatory testing was only necessary for participants who received reactive results from an HIVST. However, there were instances where a few participants with non-reactive results insisted on being re-tested. The peer educator and outreach teams provided ongoing education to instill confidence in HIVST users. In the in-depth interviews, reasons expressed for not using the kits included low risk perception, fear of results, a lack of knowledge about HIVST, lack of faith in oral kits that don't require blood, and a preference for facility-based testing and physicians. The tracking of secondary distribution was mentioned as another difficulty. Peer educators distributed kits to their peers for secondary distribution and promoted secondary distribution as a way for people to learn their HIV status. However, receiving feedback from MSM who obtained HIVST kits via secondary distribution routes remained an ongoing challenge. Peers attempted to track and follow-up with their networks and encourage them to access prevention or treatment options after testing.

Some participants stated that during the pandemic, access to kits was hampered by COVID-19 control measures, as well as by the dread of numerous stressors brought on by pandemic-related interruptions. In order to maintain continuity in the provision of services to the community, "cyber-peer educators" replaced one-on-one peer educators in online sessions. During the pandemic, difficulties included some peer educators leaving the city and being unable to return for months, which had an adverse effect on the distribution of HIVST and routine testing.

### Coverage

A total of 8,764 MSM utilized HIVST, of whom 3,569 were from Mombasa, 2,080 from Kiambu, and 3,115 from Kisumu. One-fourth of these men were connected to the intervention through online communities. With the exception of the COVID-19 period, when frequent testers were also given HIVST kits, infrequent and non-testers were the major target population for HIVST kit distribution. Distribution of 67% of the kits went to infrequent testers and non-testers.

### Acceptability and quality

MSM demonstrated a good opinion of the intervention through their active participation in the project. MSM-led CBOs were in control of the intervention, therefore they took a leading role in its conception, implementation, monitoring, and evaluation. As a result, the project fostered a sense of communal ownership and responsibility.

The analysis of the in-depth interviews revealed that the participants had a favorable opinion of the intervention. Participants, including those who did not use HIVST kits, expressed that they were aware of HIVST and had learned about it from peer educators, social media, and the DICs. The majority of participants during the COVID-19 period said that they continued to get information via social media (such as WhatsApp groups and Facebook pages) in the form of demonstration films and details on where to get kits. A significant number of participants thought the material was good and conveyed well, although some thought peer educators were less able to give precise details on linkages following testing. There were recommendations for further training of peer educators in effective linkages and referrals.

Additionally, more MSM who used HIVST tested regularly than those who did not use HIVST at the study's endline. According to the PBS findings, 82% of HIVST users had a recent test during the last three months, compared to 58% of non-users. Since HIV test results are confidential, the participants regarded HIVST as a way to decrease the number of men in their community who do not know their HIV status and link them to treatment.

*I think the self-test kit has also… it is also going to at least reduce the number of people who don’t know their HIV status and it will then help the MSM community at least to access ART and that will also reduce the HIV infection within this community of MSM*. [Kevin, Kisumu]

The majority of participants admitted to feeling anxious about using the kits for the first time. Numerous factors, including the fact that HIVST was a novel technique, contributed to their unease.

*I had some challenges because, you know, using such like thing … is not an easy task, in as much as we encourage ourselves and lie to ourselves that we are okay and healthy; it is very important that you know your status. You never know when you’ll ruin your partner’s life…. You might be willing to know your status but your partner is not willing and even discouraging you from testing. And this gets you nervous.* [Mchwa, Mombasa]

However, initial apprehension and worry subsided over time, and the majority of participants grew accustomed to using the kits and described it as a straightforward technology.

*I was using it alone [the first time] so I was sort of panicking. Well, afterwards, I became more confident about using it. Then, just that the thing; it’s so simple.* [Joe, Kiambu]

In the polling booth survey, over one-third of participants (34%) said they would prefer HIVST, even though 57% of individuals continued to choose rapid HIV testing the next time they tested. Preferences for HIVST included the capacity to maintain anonymity and increase choice, the convenience it provided under COVID-19 constraints, and its utility for secondary distribution and partner testing, as expressed in the quotations below:

*It is very private which makes someone very comfortable, it even takes away some of your fears. You know there are some people who cannot go for HIV testing, yeah, not because … they do not want; they are just afraid of the person on the other side of the table, someone can tell you that I am positive… And it is your status. You have to deal with it. Maybe the clinical officer is your, is the guy you have been fighting over with for another sexual partner … or maybe he is a relative, or maybe he is something close to you, maybe your neighbor, so that privacy really matters a lot.* [Willy, Kisumu]

*Interviewer: Where-where have you done the tests?*
*Respondent: At home…. [and] at my partner’s house…. Like I said, the social distancing. And also if you don’t have a mask you cannot access any facility at times. You don’t have cash to buy the mask so at times it becomes so hard. And also you know, now that we are at home, life in the city has become so hard due to this COVID, because our clients have reduced; so back at home you just have to (pauses) yeah.”* [Kings, Kisumu, Endline]
*There is one who is a friend of mine and is also an MSM and ah doesn’t have, I noticed doesn’t come to any DIC and doesn’t mingle in any DIC because I think he has a phobia for places like this and so I gave that one to him and then I gave one to a lover of mine who has not yet come*. [Vinny, Mombasa]

At the endline, the PBS data showed prevention services had reached 90% of participants who had ever used HIVST, compared to 76% of people who had never used HIVST. At endline, 84% of participants had been linked to care and treatment with significant improvement among virtual peers from 71% at baseline to 93% at endline. Even during COVID-19, when CBO/DIC visits were low, the majority of participants described the peer educator's role in routine follow-up as one of continual emotional support and as an essential component of community-based care.

*First of all they [peer educators] asked if I read the instructions well and tested alone or with a partner, how I was feeling. What was the result? After the result what happened? Yeah things like that, they were just asking about what happened, I told them it was good.* [Feshal, Kisumu]
*That follow-up makes you feel like, yeah, there are people outside who are caring and they just want to know how you are doing health wise.* [Coach, Mombasa]

***Effectiveness*.** When compared to baseline, the PBS data showed that the total uptake of HIVST rose noticeably at endline. At endline, 47% of MSM from both physical and virtual sites reported using HIVST in the previous three months (31% at baseline). Virtual peers showed significant improvement, increasing from 25% to 51% at baseline and endline, respectively. With an overall rise from 63% of MSM getting treatment in the past three months at baseline to 73% at endline, access to services from the project intervention also demonstrated an improvement. As the proportion of people living with HIV rose from 13% at baseline to 19% at endline, the intervention also resulted in new diagnoses.

## Discussion

The introduction of HIVST in MSM-led HIV prevention programmes was feasible with high acceptability. The involvement of the MSM community in the design, implementation, and evaluation of the intervention was a key factor to the intervention success. The HIVST intervention was a jointly developed project that was integrated into existing MSM-led organizations, with communities taking the lead in the intervention's planning, implementation, and assessment. Engagement of virtual peer educators showed viability in reaching MSM not connected to actual programme spaces. Our results demonstrated that, utilizing virtual peer educators enhanced reach and uptake of both HIVST kits and other HIV prevention and treatment services among MSM. Due to this, online users began to use HIVST kits and other services more frequently, which provided support services during the COVID-19 pandemic ( Odinga *et al.*, 2020). During the pandemic, service provision was sustained, prolonged, and adapted to meet the emerging needs of the community. The HIVST intervention provided a workable and scalable strategy for service delivery, demand generation, and linkages to other HIV prevention and treatment programmes.

The process evaluation showed good fidelity, with the planned actions for mobilization, demand generation, service delivery, and linkages carried out according to schedule, and necessary adaptations made to respond to the pandemic. This may be attributable to the study's co-creation, in which MSM-led groups and community members played a leading role in the design, planning, implementation, monitoring, and assessment. These findings support research from a South African study on the importance of co-creating HIVST delivery modalities with key stakeholders in order to improve men’s engagement with HIV services ( Mashamba-Thompson *et al.*, 2022). In order to maximize the results of any study, it is crucial that key stakeholders including researchers, service providers, and service consumers actively participate in co-ideation, co-design, co-implementation, and co-evaluation.

The HIVST intervention was carried out with neither adverse incident nor major problems. With the establishment of a centralized follow-up mechanism for HIVST users, the virtual and physical peer educators assumed a center stage in the distribution of the HIVST kits, whose users anticipated to be linked for either HIV prevention or treatment services under the intervention design. The key reason this did not happen in some cases was that MSM who have not disclosed their identities did not want to be known by the programmes, as seen in other studies exploring barriers to care among MSM ( Mashamba-Thompson *et al.*, 2022; Okall *et al.*, 2014). Programmes and researchers should work to ensure that MSM-friendly services are provided and are customized to their needs.

A large number of MSM used HIVST kits during the intervention. The programmes were able to reach previously unreached MSM due to the involvement of virtual peer educators. According to studies, MSM are increasingly using online dating services to find sexual partners ( Stahlman *et al.*, 2015). A relationship between using virtual sites and increased HIV risk was found in a cross-sectional bio-behavioural study done in Kenya ( Bhattacharjee *et al.*, 2020). Therefore, it might be helpful for programmes to better understand the experiences of MSM using virtual sites and create interventions catered to their particular needs. Research on virtual indirect secondary distribution of HIVST via social media platforms to MSM in China has shown feasibility in reaching MSM with a history of non-testing and similar outcomes in linkages to care as participants reached directly through social media sites ( Li *et al.*, 2021), providing additional modalities to reach infrequent and non-testers.

The HIVST intervention was well-received since the service users (MSM) thought the technology was practical, private, confidential, and offered options for testing. Our findings are consistent with those of other studies, which demonstrate that HIVST is practical, private, and discreet and aids in reducing stigma related to facility-based testing ( Dirisu *et al.*, 2020; Figueroa *et al.*, 2015; Young *et al.*, 2014). The PHDA regional coordinators provided ongoing technical assistance to the programmes in order to improve quality. Additionally, they took part in the monthly meetings where progress was discussed and course-corrections, such as those made during the COVID-19 pandemic, were made as a team. The intervention saw an increase in newly diagnosed MSM, an improvement in service availability, and a rise in HIVST use. The results of this study are consistent with those of other studies which have demonstrated a high uptake of HIVST ( Girault *et al.*, 2021).

## Limitations

The findings from our process evaluation rely on self-reported data, which may be impacted by desirability biases. Process notes, such as meeting minutes, might also be subject to desirability and recall bias. However, our use of multiple data sources to triangulate findings and the involvement of community researchers and key stakeholders in the design, implementation, and analysis likely worked to minimize these biases.

## Conclusion

The introduction of HIVST in MSM-led HIV prevention programmes was feasible with high acceptability. The involvement of the MSM community in the design, implementation, and evaluation of the intervention was a key factor to the intervention success. While gaps remain in reaching MSM in virtual places, the use of virtual peer educators was successful and warrants scale up. Global pandemics, such as COVID-19, demand that programmes remain flexible in their approach. Efforts must be made to encourage clinical follow-up, but this is crucially dependent on making sure that MSM-friendly services are offered.

## Data Availability

As all the datasets involve sensitive data from criminalized and stigmatized population, data will not be made publicly available. Data may be made available from the corresponding author on reasonable request and approval from the community organizations and study teams. University of Manitoba: Data collection tools. https://doi.org/10.34990/FK2/TZNLRV. This project contains the following extended data: Data collection tools Data are available under the terms of the Creative Commons Zero "No rights reserved" data waiver (CC0 1.0 Public domain dedication).
