# Supplementary material for: Expanding options for HIV testing: A process evaluation of a community-led HIV self-testing intervention among men who have sex with men in Kenya
Source: Gates Open Res. 2024 Mar 25;7:127. Originally published 2023 Oct 20. [Version 2] doi: 10.12688/gatesopenres.14819.2 (PMC11259586; doi:10.12688/gatesopenres.14819.2)
Supplement: Peer review history

# Reviewer report: Reviewer response for version 2

**DOI:** 10.21956/gatesopenres.16889.r36708

**Published:** 2024-07-19

**Authors:** Christina Misa Wong

**Affiliations:** 1 Independent consultant, Singapore, Singapore

Thank you for giving me the opportunity to review this manuscript which was well written. This was a process evaluation of a HIV self-testing intervention using a mixed methods design. The authors describe a  comprehensively carried out intervention with data collection captured at various points during the intervention and with different groups. The results of this evaluation are very important in the field of HIV self testing and how HIV testing can be increased among MSM. The study is especially commendable for partnering with the community and engaging community members in different aspects of the intervention and evaluation design as well as in data collection and interpretation.

 There are no major comments. Just a minor comment if the authors could provide a description of how the community researchers were selected and how many there were. Thank you.

Is the work clearly and accurately presented and does it cite the current literature?

Yes

If applicable, is the statistical analysis and its interpretation appropriate?

Not applicable

Are all the source data underlying the results available to ensure full reproducibility?

No source data required

Is the study design appropriate and is the work technically sound?

Yes

Are the conclusions drawn adequately supported by the results?

Yes

Are sufficient details of methods and analysis provided to allow replication by others?

Yes

Reviewer Expertise:

HIV self testing, HIV prevention among MSM, HIV care and treatment, PrEP acceptability, gender based violence.

I confirm that I have read this submission and believe that I have an appropriate level of expertise to confirm that it is of an acceptable scientific standard.

# Reviewer report: Reviewer response for version 2

**DOI:** 10.21956/gatesopenres.16889.r37215

**Published:** 2024-07-19

**Authors:** Mamadu Baldeh

**Affiliations:** 1 University of Sierra Leone College of Medicine and Allied Health Sciences (Ringgold ID: 256445), Freetown, Western Area, Sierra Leone; 2 Medical Research Council Unit, The Gambia, London School of Hygiene and Tropical Medicine Faculty of Infectious and Tropical Diseases (Ringgold ID: 218289), London, England, UK

This implementation science study on community-led HIV self-testing for MEM in Kenya is well-designed and provides important contributions to overall HIV care for minority groups, particularly in challenging settings. 

 Minor comment for consideration:

 1. Please elaborate on the total HIV positives reported as a result of study intervention and the steps taken to facilitate initiation of treatments (during the study)

 2. How was access to and retention in service considered for those who tested positive during the study? 

 3. How can access to HIVST kits be made easy after the study concludes for this minority group?

 4. How can retention in service be improved for a minority group like MSM in a highly stigmatized setting?

Is the work clearly and accurately presented and does it cite the current literature?

Yes

If applicable, is the statistical analysis and its interpretation appropriate?

Not applicable

Are all the source data underlying the results available to ensure full reproducibility?

No source data required

Is the study design appropriate and is the work technically sound?

Yes

Are the conclusions drawn adequately supported by the results?

Yes

Are sufficient details of methods and analysis provided to allow replication by others?

Yes

Reviewer Expertise:

Infectious disease epidemiology, Health systems research, HIV-&-Non-communicable disease epidemiology, Infectious disease diagnostics at Point-of-care.

I confirm that I have read this submission and believe that I have an appropriate level of expertise to confirm that it is of an acceptable scientific standard.

## References

[1] : Expanding options for HIV testing: A process evaluation of a community-led HIV self-testing intervention among men who have sex with men in Kenya. *Gates Open Research* .2024;7: 10.12688/gatesopenres.14819.2 10.12688/gatesopenres.14819.2 PMC1125958639035469

# Reviewer report: Reviewer response for version 1

**DOI:** 10.21956/gatesopenres.16146.r35477

**Published:** 2023-12-21

**Authors:** Cedric Bien-Gund

**Affiliations:** 1 Division of Infectious Diseases, Department of Medicine, University of Pennsylvania, Philadelphia, Pennsylvania, USA

This manuscript investigates the implementation of a community-led HIVST intervention focused on MSM in Kenya. I appreciated the good use of mixed methods to describe process outcomes.

 A few minor comments:

  

- Could the authors provide a little more detail on how peer educators were chosen, and who they were? Were they also MSM?
- In the acceptability and quality section, with the qualitative data, it would be helpful to know a little more context for each of the chosen quotations. Are they first time testers? Infrequent testers? How old?

Is the work clearly and accurately presented and does it cite the current literature?

Yes

If applicable, is the statistical analysis and its interpretation appropriate?

Not applicable

Are all the source data underlying the results available to ensure full reproducibility?

No source data required

Is the study design appropriate and is the work technically sound?

Yes

Are the conclusions drawn adequately supported by the results?

Yes

Are sufficient details of methods and analysis provided to allow replication by others?

Partly

Reviewer Expertise:

HIV prevention, HIV self-testing, sexual and gender minorities

I confirm that I have read this submission and believe that I have an appropriate level of expertise to confirm that it is of an acceptable scientific standard.

# Reviewer report: Reviewer response for version 1

**DOI:** 10.21956/gatesopenres.16146.r35481

**Published:** 2023-11-22

**Authors:** Sandra Hsu Hnin Mon

**Affiliations:** 1 Big Data Institute, Nuffield Department of Medicine, University of Oxford, Oxford, England, UK

Thank you for the opportunity to review this manuscript by Melon et al. The study described here is well-designed and contributes highly relevant implementation science data on HIVST for MSM in Kenya. The study is valuable with its community-partnered approach and its utilization of the virtual space for demand creation and peer education – the latter has particular importance for other SSA settings where sexual and gender minorities face disproportionate stigma.

 Provided below in good faith are some major and minor recommendations, which may strengthen this manuscript:

**Major comments:**

1. **Overall: **Please clarify if all participants in this analysis were exclusively cisgender MSM. If not, please include a clear statement whether transgender women were included under the “MSM” acronym used in this manuscript. Transgender women have distinct behavioral and HIV risk profiles compared to cis men, and a disclaimer would be helpful for reader clarity and to ensure appropriate inferences for future research and/or programmatic work.
2. **Overall: **Please clearly define “hotspot” as a high-traffic or popular venue/platform, or consider a different term altogether. “Hotspot” may have negative connotations with respect to sexual/gender minority spaces or HIV transmission.
3. **Results:** If possible, please include a sociodemographic table of the participant population and highlight if certain factors were conducive (or not) to increased uptake, etc.
4. **Results:** For better assessment of baseline vs. endline impact, please consider standardizing the reporting of cross-sectional proportions, especially in the Acceptability and Effectiveness subsections. A table could also be helpful here.

**Minor comments:**

1. **Methods:** It is not clear whether pre- and post-HIVST counselling was actively provided, particularly to MSM participants in the community-based delivery channels or those who did not consent to clinical follow-up. If this counseling was provided as part of the service delivery component, please indicate so.
2. **Results, current Table 1: **Do consider adding a column of negative findings (or “current barriers”), as these may also provide important context for future HIV control efforts for MSM in Kenya.

Is the work clearly and accurately presented and does it cite the current literature?

Yes

If applicable, is the statistical analysis and its interpretation appropriate?

I cannot comment. A qualified statistician is required.

Are all the source data underlying the results available to ensure full reproducibility?

Partly

Is the study design appropriate and is the work technically sound?

Yes

Are the conclusions drawn adequately supported by the results?

Yes

Are sufficient details of methods and analysis provided to allow replication by others?

Yes

Reviewer Expertise:

Infectious disease epidemiology, HIV epidemiology, health and human rights, sexual and gender minorities

I confirm that I have read this submission and believe that I have an appropriate level of expertise to confirm that it is of an acceptable scientific standard, however I have significant reservations, as outlined above.

# Response

**Published:** 2024-03-20

**Authors:** Lisa Lazarus

**Affiliations:** University of Manitoba, Winnipeg, Manitoba, Canada

We thank the reviewer for their comments and have provided detailed responses to each point below:

**1. Overall: **Please clarify if all participants in this analysis were exclusively cisgender MSM. If not, please include a clear statement whether transgender women were included under the “MSM” acronym used in this manuscript. Transgender women have distinct behavioral and HIV risk profiles compared to cis men, and a disclaimer would be helpful for reader clarity and to ensure appropriate inferences for future research and/or programmatic work.

**Response: **We thank you for this important comment. We have clarified that the eligibility criteria included participants who identified as men who have sex with men.

**2. Overall: **Please clearly define “hotspot” as a high-traffic or popular venue/platform, or consider a different term altogether. “Hotspot” may have negative connotations with respect to sexual/gender minority spaces or HIV transmission.

**Response: **The term ‘hotspot’ has been replaced with ‘sites’ in the entire document to address the negative connotation.

**3. Results:** If possible, please include a sociodemographic table of the participant population and highlight if certain factors were conducive (or not) to increased uptake, etc.

**4. Results:** For better assessment of baseline vs. endline impact, please consider standardizing the reporting of cross-sectional proportions, especially in the Acceptability and Effectiveness subsections. A table could also be helpful here.

**Response: **In response to point 3 and 4, we have clarified on page 12 that sociodemographic data and epidemiological analyses have been published elsewhere: “participant data is presented in more detail elsewhere: for integrated biological and behavioural assessment data see Shaw et *al*., 2023; for network data see Lazarus et *al*., 2022; for interview data see Thomann et *al*., 2022)”.

**Minor comments:**

**1.Methods:** It is not clear whether pre- and post-HIVST counselling was actively provided, particularly to MSM participants in the community-based delivery channels or those who did not consent to clinical follow-up. If this counseling was provided as part of the service delivery component, please indicate so.

**Response: **-Thank you for this question. While pre- and post-test counselling can be a challenge for HIVST, we clarify on page 10 that: “A coordinated follow-up system was put in place a few months into the intervention to efficiently help connect the recipients of HIVST kits to prevention and treatment services. For MSM who had consented to be followed, the clinical team (HTS providers and clinicians) made phone calls and sent text messages, with the assistance of peer educators. Peer educators and peers supported follow-up for MSM not consenting to be followed by health care providers. Referral cards with clinical contacts were given out along with HIVST kits so that participants could call or text the clinical team for more information or for referrals. To facilitate linkages to services, accompanied referrals were also offered”.

**2. Results, current Table 1: **Do consider adding a column of negative findings (or “current barriers”), as these may also provide important context for future HIV control efforts for MSM in Kenya.

**Response: **-Table 1 (p.12-13) includes both “positive” and “negative” findings under the heading “Findings”.
